# The regulated cell death at the maternal-fetal interface: beneficial or detrimental?

**DOI:** 10.1038/s41420-024-01867-x

**Published:** 2024-02-26

**Authors:** Huan Chen, Yin Chen, Qingliang Zheng

**Affiliations:** Prenatal Diagnosis Center, The Eighth Affiliated Hospital, Sun Yat-sen University, 3025# Shennan Road, Shenzhen, 518000 P.R. China

**Keywords:** Cell death, Infertility

## Abstract

Regulated cell death (RCD) plays a fundamental role in placental development and tissue homeostasis. Placental development relies upon effective implantation and invasion of the maternal decidua by the trophoblast and an immune tolerant environment maintained by various cells at the maternal-fetal interface. Although cell death in the placenta can affect fetal development and even cause pregnancy-related diseases, accumulating evidence has revealed that several regulated cell death were found at the maternal-fetal interface under physiological or pathological conditions, the exact types of cell death and the precise molecular mechanisms remain elusive. In this review, we summarized the apoptosis, necroptosis and autophagy play both promoting and inhibiting roles in the differentiation, invasion of trophoblast, remodeling of the uterine spiral artery and decidualization, whereas ferroptosis and pyroptosis have adverse effects. RCD serves as a mode of communication between different cells to better maintain the maternal-fetal interface microenvironment. Maintaining the balance of RCD at the maternal-fetal interface is of utmost importance for the development of the placenta, establishment of an immune microenvironment, and prevention of pregnancy disorders. In addition, we also revealed an association between abnormal expression of key molecules in different types of RCD and pregnancy-related diseases, which may yield significant insights into the pathogenesis and treatment of pregnancy-related complications.

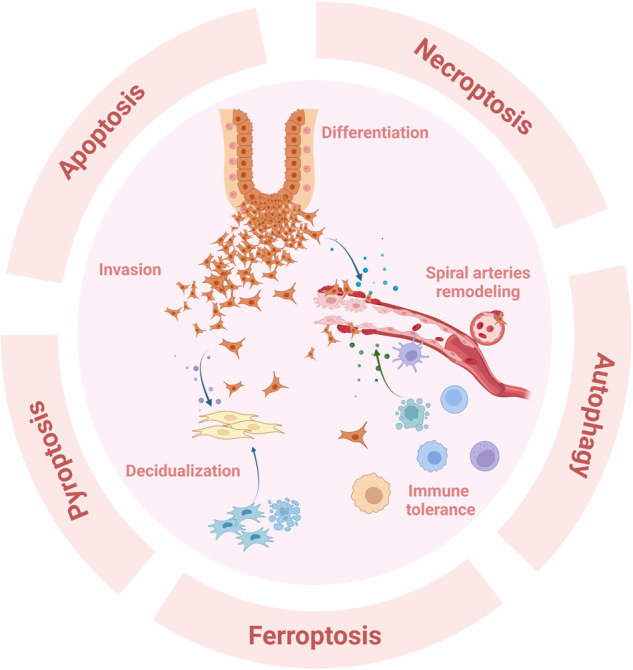

## Facts

1. Multiple forms of regulated cell death exist at the maternal-fetal interface.
2. Trophoblast cells undergo various forms of RCD, which can influence their proliferation, invasion, and uterine spiral artery remodeling.
3. RCD is implicated in the regulation of decidualization.
4. Abnormal RCD levels have been detected in placenta and decidua tissues of patients with pregnancy complications.

## Open Questions

1. What are the different types of cell death present in various cell types at the maternal-fetal interface?
2. What is the positive role of regulated cell death in normal pregnancy processes?
3. How does dysregulated regulated cell death contribute to the pathogenesis of pregnancy complications?
4. How do different forms of regulated cell death interact and what are the underlying molecular mechanisms involved in their regulation?

## Introduction

The placenta provides oxygen and nutrients to the fetus during pregnancy while removing carbon dioxide and other waste products. The growth of the placenta are precisely regulated, interactions between immune cells, decidual stromal cells (DSCs) and trophoblasts at the maternal-fetal interface form a vast network of cellular connections that support the early development of the embryo. During decidualization, ESCs transform into DSCs, undergoing morphological and functional changes [1]. DSCs secrete a variety of cytokines to communicate with other cells and provide essential nutrients for embryo implantation and placental development [2]. A continuous supply of nutrients is essential to support the growth and development of the fetus and placenta.

Placental trophoblasts invade the maternal decidua and uterine basal layer, remodeling the uterine spiral arteries to ensure adequate oxygen supply for fetal development, which is crucial for a successful pregnancy [3]. Decidual immune cells coordinate trophoblast invasion and spiral artery remodeling while regulating the immune balance of the maternal-fetal interface, making the interface tolerant to semi-allogeneic fetuses and maintaining local defense against pathogen infection [4]. These processes are controlled by multiple cellular mechanisms such as regulated cell death (RCD). Cell death is an inevitable and irreversible phenomenon that occurs during the growth and development of organisms. According to the updated guidelines by the Committee on Cell Death Nomenclature in 2018, cell death is classified into 12 types based on its morphological, biochemical and functional characteristics [5]. RCD is the basis of many physiological features and plays an indispensable role in embryonic development, organ formation, resistance to pathogen invasion, inhibition of rapid tumor cell proliferation, and the maintenance of homeostasis [6].

**Fig. 1:**
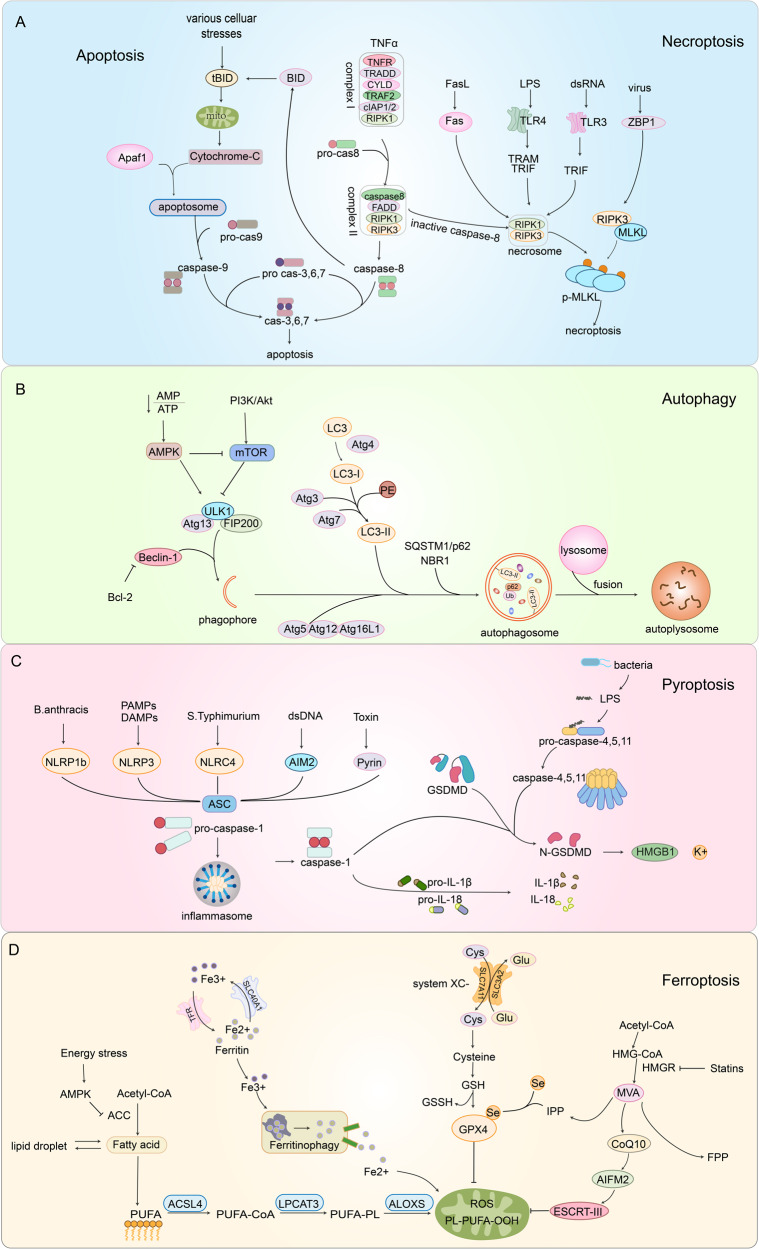
Major molecular mechanisms of different types of regulated cell death. **A** In apoptosis, the intrinsic pathway is mainly mediated by mitochondria. TNFα can stimulate the extrinsic pathway and induce apoptosis (left). If caspase-8 activity is inhibited, RIPK1 forms necrosomes with RIPK3 and MLKL, thereby triggering necroptosis (right). **B** Autophagy begins with the formation of phagophores. This process is regulated by ULKL and Beclin-1. The ATG12-ATG5-ATG16L1 complex and LC3-II recruit loads to cargo receptors, which is essential for the process of phagophore expansion to generate autophagosomes. **C** In the classical pyroptosis pathway, when cells are exposed to external stimuli, activated caspase-1 can cleave GSDMD to produce an N-terminal of GSDMD, which can form holes in the cell membrane to release mature IL-1β and IL-18 and induce pyroptosis. In the non-classical pyroptosis pathway, GSDMD is cleaved by caspase-4, -5, and -11. Pyroptosis can also trigger the release of HMGB1 and K^+^. **D** Iron accumulation and lipid peroxidation are important factors that trigger reactive oxygen species (ROS) production and ferroptosis. The ACSL4-LPCAT3-ALOXs pathway enables the production of phospholipid hydroperoxide (PLOOH) from polyunsaturated fatty acids (PUFAs), which is oxidized from fatty acids. In the most studied antioxidant system, the Xc-system-GSH-GPX4, GPX4 can scavenger oxygen free radicals and inhibit ferroptosis. Other antioxidant systems, such as CoQ10-AIFM2 and ESCRT-III membrane repair systems, also play important roles in inhibiting lipid peroxidation.

Currently, the most extensively studied types of RCD are apoptosis, autophagy, necroptosis, pyroptosis and ferroptosis. Apoptosis plays a vital role in normal eukaryotic development and maintains homeostasis [7]. According to internal or external stimuli and the involvement of various adapter proteins and initiator caspases, apoptosis classified into two canonical pathways: extrinsic and intrinsic pathways (Fig. 1A). Autophagy is a primary self-degrading cellular process that accelerates metabolism and is ubiquitous in eukaryotic cells. Under stress conditions, such as energy or nutrition shortages, the level of autophagy increases because most cells have a low level of autophagy to remove superfluous and damaged organelles to maintain cellular homeostasis. Excessive autophagy leads to cell death [8] (Fig. 1B). Unlike apoptosis and autophagy, necroptosis, another type of regaluted cell death, is triggered by the phosphorylation of mixed-lineage kinase-like (MLKL) by receptor-interacting kinase-3 (RIPK3) [9]. Necroptosis can be triggered by multiple stimuli and is primarily mediated by cytokines, toll-like receptors (TLRs) and nucleic acid receptors. Different signaling pathways contribute to RIPK3 activation in several ways. TNFα-induced MLKL activation is dependent on RIPK1 [10]. When RIPK1 is absent, interferons induce the formation of the Z-DNA binding protein-1 (ZBP1)-RIPK3 complex that induces necroptosis mediated by MLKL [11] (Fig. 1A). Pyroptosis is a form of RCD activated by inflammasomes and plays an important role in inflammation and immunity [12]. There are some morphological similarities between pyroptosis and necroptosis such as plasma membrane rupture. However, pyroptotic cells show swelling prior to membrane rupture, with many bubble-like projections appearing on the surface of the cell membrane [13] (Fig. 1C). Ferroptosis, a newly identified type of regulated cell death first described in 2012, is a unique reactive oxygen species (ROS)- and iron-dependent mode of cell death [14]. The major cytological changes in ferroptosis mainly manifest as mitochondrial abnormalities. Lipid peroxidation and the accumulation of ROS are the main reasons resulting in ferroptosis [15] (Fig. 1D). There is extensive crosstalk during different regulated cell death pathways. PANoptosis is a coordinated cell death pathway involving pyroptosis, apoptosis and necroptosis [16]. AIM2, Pyrin and ZBP1 forms a complex to drive PANoptosis and provide host protection in response to viral infection [17].

The effects of different RCD at the maternal interface may vary. Some are conducive to maintaining the normal physiological processes of the body, whereas others cause disorders and adverse consequences. This study reviews the five most extensively studied types of RCD occurring in trophoblasts, DSCs and various decidual immune cells at the maternal-fetal interface during pregnancy and their biological function effects involving trophoblasts activity, decidualization, and maintenance of immune homeostasis. Furthermore, the impact of RCD on various complications during pregnancy, such as preeclampsia, gestational diabetes mellitus and spontaneous abortion, have also been discussed.

## Involvement of RCD at the maternal-fetal interface in pregnancy

During pregnancy, the placenta undergoes remarkable changes in morphology and function to modulate maternal physiology and metabolism [18]. Cells at the maternal-fetal interface are multitudinous and are usually divided into trophoblasts, decidual immune cells and DSCs. Exposure to a relatively hypoxic environment is essential for placental development and successful trophoblast proliferation during early pregnancy [19]. However, a severely hypoxic environment has the opposite effect. Hypoxia [20], external pollutants [21], pathogens [22], homeostatic imbalances [23] and other factors [24] may cause trophoblasts undergo various cell death modes. Some of types of RCD are needed for embryo implantation and development [25], whereas some may damage the function of trophoblasts and even cause adverse outcomes like preeclampsia [26]. Therefore, it is essential to understand the effects of different types of RCD on the biological functions of trophoblasts.

### Effect of RCD on trophoblast differentiation

Mononucleated cytotrophoblasts differentiate along with losing their cell boundaries, and fuse to form syncytiotrophoblasts, which undergo tightly regulated differentiation [27]. Transcriptomic and pathway analyses revealed that autophagy-related genes were upregulated during trophoblast differentiation and fusion. The autophagy marker (LC3B) is predominantly detectable in syncytiotrophoblasts of the human placenta during the first trimester, indicating that autophagy is involved in regulating trophoblast fusion [28]. Enhanced autophagic flux occurs in trophoblast stem cells and cytotrophoblasts [29]. Autophagy promotes trophoblast differentiation by regulating galectin-4 expression to promote cell-cell adhesion [30]. The fine-tuning of autophagy activation is crucial for promoting cell survival during trophoblastic syncytialization [31, 32]. Excess apoptosis induced by autophagy or other factors (such as heme) can inhibit trophoblast fusion, leading to adverse consequences [33, 34]. However, appropriate apoptosis is essential for triggering syncytial fusion. A subset of cytotrophoblasts, influenced by DNA transcription factors, initiates the expression pathway of apoptosis-related proteins that are necessary for syncytial fusion [35]. Primary human trophoblasts show lower levels of apoptosis in syncytiotrophoblasts than in cytotrophoblasts [36]. Pro-apoptotic Mtd/Bok is also involved in regulating proliferation of trophoblast cell [37]. Moreover, decreased apoptosis and autophagy have been observed in the placentas of women with gestational diabetes mellitus and large-for-gestational-age infants [38]. Thus, trophoblast differentiation relies on moderate trophoblast autophagy and apoptosis. Pathological pregnancy may occur if the dynamic balance is disrupted.

The loss of E-cadherin (CDH1)-stained cell boundaries between neighboring cells is an indicator of trophoblast fusion, as this adhesion protein is only expressed in the plasma membrane of cytotrophoblasts, but not in syncytiotrophoblasts [39, 40]. CDH1-stained intercellular boundaries and strong p-MLKL expression have been observed in primary trophoblast cells after treated with ceramide, indicating the activation of necroptosis pathway. Transcription factor GCM1 expressed in cytotrophoblasts before fusionis required for syncytiotrophoblast formation. Its expression level decreases in primary trophoblasts when necroptosis occurs [41]. Thus, necroptosis may interfere with cytotrophoblast differentiation.

**Fig. 2:**
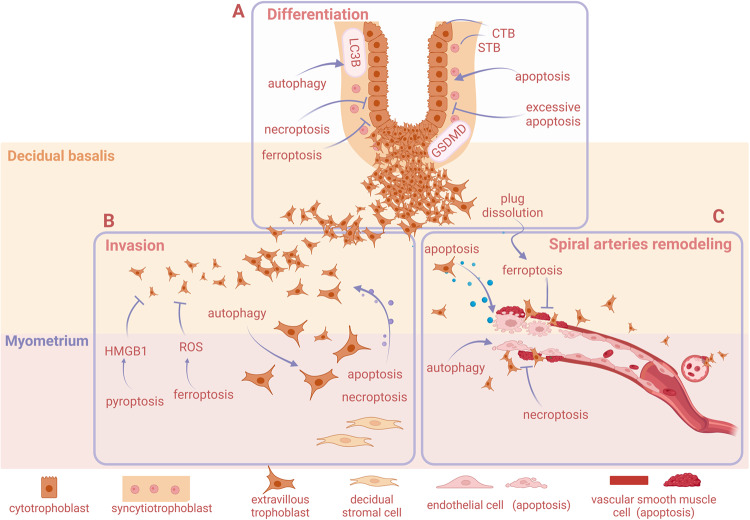
Regulated cell death affects trophoblast differentiation, invasion and vascular remodeling. **A** Apoptosis can promote the differentiation and fusion of cytotrophoblast cells to form syncytial trophoblasts; however, excessive apoptosis inhibits this differentiation process. Necroptosis can also affect trophoblast differentiation. Inhibition of ferroptosis can promote fusion. Pyroptosis- and autophagy-related proteins have been detected in syncytial trophoblasts. **B** Apoptosis and necroptosis in ESCs contribute to trophoblast invasion. Deficiency in autophagy or apoptosis in trophoblast cells can lead to shallow invasion. Pyroptotic cells can release HMGB1, which inhibits trophoblast invasion. Ferroptosis inhibits trophoblast invasion. **C** Trophoblast cells secrete cytokines to induce apoptosis of vascular endothelial smooth muscle cells and epithelial cells to promote arterial remodeling. Arterial remodeling also requires trophoblast autophagy. Necroptosis lead to failures in spiral artery remodeling. Trophoblastic plug dissolution is prone to trigger ferroptosis, which may affect spiral artery remodeling. Created with BioRender.com.

Low-dose inhibitor of GPX4 (RSL3) leads to trophoblastic dysfunction, which reduces the population of cytotrophoblasts to form syncytiotrophoblasts, but this effect is reversed by the ferroptosis inhibitor (ferrostatin-1) [39]. GSDMD is predominantly located at the apical surface of syncytiotrophoblasts in the villous trophoblast layer but rarely in cytotrophoblast cells, marked staining of caspase-1 in the trophoblast layer has been observed in syncytiotrophoblasts [42] (Fig. 2A). These reports indicated that ferroptosis and pyroptosis may play a role in regulating syncytiotrophoblast differentiation. The differentiation of trophoblast cells is influenced by different RCD. Hence, the current study suggests that RCD plays an important role in the differentiation process of trophoblast cells, with moderate autophagy and apoptosis favoring trophoblast differentiation and necroptosis and ferroptosis playing a negative role. However, the underlying mechanisms and crosstalk between different death pathways still require further investigation.

### Effect of RCD on trophoblast invasion

Extravillous trophoblasts (EVTs) invade the decidua and part of the myometrium, thereby anchoring the fetus to the uterus. This process requires some types of RCD which mainly promote trophoblast cell invasion [43, 44]. Trophoblasts secrete factors that induce apoptosis of endometrial epithelial cells, facilitating the implantation of blastocysts into the endometrial stroma [45]. Hypoxia-induced autophagy in placental trophoblast cells can counteract ox-LDL-induced apoptosis, preserving trophoblast balance and enhancing the invasion of EVT cells [46]. Transmission electron microscopy has shown that autophagosomes are distributed on the surface of microvilli in normal villus tissue, and LC3B is present in EVT cells at deeper site of decidua basalis. Although the viability and proliferation of wild-type and autophagy-deficient EVT cells are similar under conditions of hypoxia, the invasion depth of autophagy-deficient EVT cells is significantly shallower [47]. Autophagy induced by chorionic villus-derived mesenchymal stem cells promotes trophoblast proliferation and enhances their invasiveness [48]. Decreased autophagy induced by Yin Yang 1 (YY1) [49] or paternally expressed gene 10 (PEG10) [50] leads to deficient trophoblast invasion, and might be involved in RSA pathogenesis. Using trophoblast-specific autophagy-related (Atg7) knockout mice, it was found that the number of trophoblasts migrating into the maternal decidua was significantly reduced, and the placentas were much smaller in knockout mice [51]. Therefore, defects in autophagy may lead to shallow trophoblast invasion.

The regulation of autophagy plays a crucial role in trophoblast cell invasion. Mitofusin-2 regulates mitochondrial autophagy, while low levels of mitofusin-2 expression increase trophoblast autophagy, which is associated with early unexplained miscarriages [52]. Excessive autophagy activation caused by decreased lei-7i expression or maternal exposure to nanoparticles, suppresses trophoblast migration and invasion [53–55].

Both apoptotic and non-apoptotic cell death mechanisms, such as necroptosis, are involved in eliminating the luminal epithelium for successful embryo invasion. While apoptosis plays a role in the initial stages of luminal epithelium loss around the embryo, it is not the primary mechanism [56]. The presence of p-RIPK1, p-RIPK3 and p-MLKL in uterine epithelial cells suggests that necroptosis may be active in these cells. Impaired necroptosis uterine epithelium correlates with subsequent failure of embryo invasion [57]. This finding suggests that necroptosis may plays a positive role in embryonic invasion.

Ferroptosis, characterized by intracellular lipid peroxidation and elevated ROS production [58], enhanced fatty acid oxidation impairs trophoblasts invasion [59]. *miR-30-5p*-mediated ferroptosis of trophoblasts reduces cell viability and invasion, which can be reversed by overexpression of SLC7A11 and Pax3 [60]. Impaired trophoblast invasiveness is associated with decreased levels of Nrf2/GPX4, which plays a role in inhibiting trophoblast ferroptosis [61]. Pyroptosis, another cell death pathway, has been observed in primary human trophoblasts under pathophysiological condition [42]. Enhanced oxidative stress or NLRP3-inflammasome-activation-induced pyroptosis inhibit trophoblast cell proliferation, migration and invasion in both in vitro and in vivo models of preeclampsia [62, 63] (Fig. 2B). Thus, in addition to apoptosis and autophagy, necroptosis has also been found to play an important role in trophoblast invasion while ferroptosis and pyroptosis tend to exert negative effects, trophoblast invasion is regulated by multiple RCD types.

### Effect of RCD on spiral artery remodeling

Remodeling of the uteroplacental spiral arteries is essential for placental development. EVT cells have a highly invasive phenotype and acquire endothelial-like characteristics, they can penetrate the uterine spiral arteries and replace maternal endothelial cells (ECs), remodeling the uterine spiral arteries into low-resistance vessels [64].

Moderate apoptosis and autophagy positively affect spiral artery remodeling. Trophoblasts secrete tumor necrosis factor-alpha-related apoptosis-inducing ligand (TRAIL) to induce the apoptosis of vascular smooth muscle cells (SMCs) [65]. SMCs and ECs can also undergo apoptosis prior to trophoblast invasion, incomplete loss of SMCs and ECs impairs the remodeling of the uterine spiral artery. A recent study indicated that atrial natriuretic peptide (ANP) promotes TRAIL expression of ESCs to facilitate spiral artery remodeling [66]. In tube-formation assays, tube structure was predominantly formed by endothelial cells rather than EVT cells when when endothelial cells were co-cultured with autophagy-deficient EVT cells [67]. Therefore, autophagy is required for EVT cells to participate in spiral artery remodeling. However, superfluous autophagic activation due to the inhibition of protein kinase C β and dysfunction of Shh signaling leads to impaired angiogenesis, which contributes to the pathogenesis of preeclampsia or recurrent miscarriage [68, 69].

SIRT3 deficiency promoted necroptosis of trophoblasts, reduces vascular endothelial growth factor (VEGF) levels and spiral artery remodeling [70]. Trophoblastic G protein-coupled receptor kinase (GRK2) induces para-vascular necroptosis and reduces the population of trophoblast giant cells at the maternal-fetal interface, leading to failure of spiral artery remodeling [71] (Fig. 2C). Trophoblasts are particularly prone to ferroptosis because of their high iron content [72]. Dissolution of trophoblastic plugs induces hypoxia/reperfusion, high levels of key molecules involved in ferroptosis appear on trophoblasts [39]. External factors like smoking can also cause ferroptosis in the placentas of pregnant women [73], triggering adverse pregnancy outcomes. Ferritin light chains (FTL) reduction during pregnancy triggered ferroptosis, disturbing uterine spiral artery remodeling [74]. As well as trophoblast differentiation and invasion, current research indicates moderate apoptosis and autophagy but not ferroptosis is beneficial for uterine spiral artery remodeling. However, further studies are needed to clarify more precise effects of ferroptosis and pyroptosis on uterine spiral artery remodeling.

### Effect of RCD on decidual immune cells

**Fig. 3:**
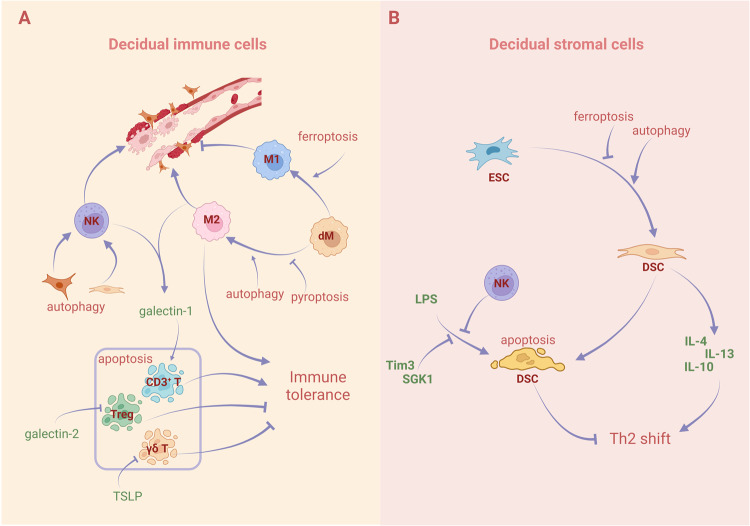
Effect of regulated cell death on decidual immune cells and stromal cells. **A** Natural killer (NK) cells can induce apoptosis of smooth muscle cells (SMCs) and endothelial cells (ECs) to promote vascular remodeling. Insufficient autophagy in extravillous trophoblasts (EVTs) or decidual stromal cells (DSCs) can affect the residence and function of NK cells. Galectin-1 secreted by NK cells and macrophages induces apoptosis of CD3^+^ T cells, while galectin-2 and TSLP inhibit the apoptosis of Treg and γδT cells respectively to maintain immune homeostasis. Ferroptosis facilitates the differentiation of macrophages into the M1 type and autophagy promotes the differentiation into the M2 type. Pyroptosis also affects the differentiation of M2 macrophages. **B** DSCs can induce the production of Th2 cytokines and promote immune tolerance. Autophagy can promote the decidualization of ESCs to DSCs but ferroptosis plays the opposite role. Lipopolysaccharide (LPS) stimulation can induce the apoptosis of DSCs, increase the secretion of Th1 inflammatory factors, and cause an inflammatory response. Decidual NK (dNK) cells, SGK1, and Tim-3 inhibit LPS-induced apoptosis. Before decidualization, DSCs also secrete pro-apoptotic molecules to induce the apoptosis of undifferentiated DSC. Created with BioRender.com.

The maternal-fetal interface harbors a diverse population of immune cells, which are crucial for fetal development and protection against viral infection. Within the decidua, natural killer (NK) cells are the most abundant immune cells in the first trimester, although the proportion of different immune cells vary throughout pregnancy [75]. Decidual immune cells undertake a series of RCD processes to maintain the immune microenvironment, and disorder of RCD can lead to pregnancy-related diseases (Fig. 3A).

### Decidual natural killer cells

The proportion of decidual natural killer (dNK) cells dynamic elevate in the first trimester and then decrease in the mild and late trimester. dNK cells have distinct transcriptional profiles and surface phenotypes compared to peripheral blood NK cells, and they play a crucial role in decidual vascular remodeling and promoting trophoblast invasion [76].

SMC and EC apoptosis induced by dNK cells is essential for decidual artery remodeling [77]. Vascular remodeling can be categorized into trophoblast-independent and trophoblast-dependent stages. Smith et al. showed that dNK are involved in the trophoblast-independent stage and proposed that remodeling requires apoptosis [77]. dNK cells from low-resistance uterine vessels exhibit a stronger ability to induce SMCs and ECs apoptosis compared to dNK cells from high-resistance vessels, highlighting the role of NK cells in vascular remodeling [78].

The proper functioning of dNK cells relies on an autophagic environment. The autophagy of DSCs, which promotes dNK cells infiltration and enrichment, is necessary for generalized decidualization [79]. dNK cells co-cultured with autophagy-inhibited trophoblasts show increased cytotoxicity, thereby decreased trophoblast proliferation and invasiveness [50]. However, it is not yet known whether dNK cells affect their functions through other RCD.

### Decidual macrophages

Decidual macrophages induce extracellular matrix degradation during vascular remodeling [77, 80]. The polarization of anti-inflammatory (M2) macrophages contributes to successful pregnancy [81]. RCD that can maintain the predominance of decidual macrophages with M2 phenotype are beneficial for pregnancy.

Oxidative stress-induced pyroptosis inhibited M2 macrophage polarization [62]. In women experiencing spontaneous preterm labor, decidual macrophages undergo pyroptosis, as evidenced by the presence of active caspase-1, GSDMD and mature IL-1β [82]. Additionally, high levels of HMGB1 secretion by decidual macrophages have been observed in decidual tissues from patients with unexplained recurrent spontaneous abortion (URSA), where it activates pyroptosis, contributing to the destruction of the maternal-fetal interface [83].

Decidual macrophages can regulate the apoptosis of trophoblasts under various conditions. Indoleamine 2,3-dioxygenase (IDO), an enzyme involved in physiological and immune regulation, is expressed in IDO^+^ decidual macrophages with an M2 phenotype. Inhibiting the IDO pathway in decidual macrophages and co-culturing them with trophoblasts results in the downregulation of proliferation- and invasion-related molecules, while promoting trophoblast apoptosis [84]. FasL in decidual macrophages mediates trophoblast apoptosis [85]. Decreased nitric oxide (NO) concentration and NOS activity in macrophages further induce trophoblast apoptosis, those apoptosis is associated with recurrent miscarriage [86].

Appropriate autophagy has been found to facilitate the adhesion, retention and M2 differentiation of decidual macrophages. In patients with URSA, the reduced residence of decidual macrophages can be alleviated by autophagy inducer rapamycin [87]. Inhibiting APOC1 promotes M1 polarization of macrophages via the ferroptosis pathway in hepatocellular carcinoma [88]. However, whether ferroptosis affects decidual macrophages polarization has not yet been studied.

### Decidual T cells

Although T cells are present in small proportions in the decidua, they play a crucial role in protecting the fetus from rejection and combating pathogenic infections. T cell apoptosis serves as a defense mechanism to prevent maternal rejection of fetal allografts [89]. Human decidual T cells have a unique glycophenotype that enables them to bind to galectin-1 [90]. Decidual macrophages and dNK cells secrete galectin-1, which induces apoptosis in CD3^+^ T cells, establishing an immune-privileged environment at the maternal-fetal interface [91]. Galectin-2 prevents the apoptosis of regulatory T cells, which play a vital role in restraining excessive immune responses and inducing immune tolerance [92]. Moreover, many other cytokines also participate in T cell apoptosis. For instance, thymic stromal lymphopoietin suppresses the apoptosis of decidual γδ T cells, while IL-10 reduces the apoptosis of decidual Treg cells [93–95].

### Decidual dendritic cells

Dendritic cells (DCs) function as a bridge between innate and adaptive immunity. It has been demonstrated that DCs are distributed around blood vessels in the human decidua and are involved in angiogenic responses, reduction of DCs results in spontaneous abortion. Immature myeloid DC (SIGN^+^ DC) subsets in human decidua promote immune tolerance and support a Th2-dominant immune response, which is beneficial for maintaining pregnancy. The number of mature myeloid CD83^+^ DCs, decreases in the decidua during decidualization [96]. The interaction between SIGN^+^ DCs and NK cells leads to a “boiling” morphology in DCs. The apoptosis of DCs prevents their further maturation and helps controling the immune stimulation of Th1 cells [97]. Regrettably, the investigation of DC subsets in the human decidua has been hindered by the paucity of DCs, posing a significant challenge to researchers in the field.

To sum up, RCD can regulate the function of different immune cells in decidua. Apoptosis and autophagy are involved in the regulation of decidual NK cells and macrophages. Pyroptosis and ferroptosis may affect the polarization of macrophages. What has been mainly reported in decidual T cells and DC cells is that apoptosis plays an important role.

### Effect of RCD on decidual stromal cells

Decidualization of the maternal endometrium is necessary for successful implantation and embryo development. Specialized DSCs are produced through the differentiation and transformation of ESCs [98]. DSCs contribute to immune tolerance by stimulating dNK cells to produce Th2 cytokines, such as IL-4, IL-13, and IL-10 [99]. Apoptosis of DSCs induced by lipopolysaccharide (LPS) increses the secretion of pro-inflammatory Th1 cytokines thereby potentially contributing to the occurrence of spontaneous miscarriage [100]. However, SGK1, Tim-3, glutamine oxidation can protect DSCs from LPS-induced apoptosis and restore their ability to secrete Th2-type cytokines [100–103]. DSCs secrete soluble pro-apoptotic factors, which can drive decidualization before embryo implantation [104]. Forkhead box O3a (FOXO3a), a highly conserved transcription factor of apoptosis-related genes, is highly expressed in the primary stromal cells [105]. Folate deficiency can impair decidualization by disrupting autophagy and decreasing apoptosis [106]. Therefore, a certain level of DSC apoptosis is necessary for proper decidualization.

DSCs harbor necroptotic machinery. Polyinosinic-polycytidylic acid (Poly[I:C])-induced DSC death is characterized by the induction the phosphorylation of MLKL, which can be reversed by Nec-1 inhibitor, but not by z-VAD-fmk. Transfection of poly(I:C) or addition of extracellular poly(I:C) triggered necroptosis of DSCs by different mechanisms [107].

Autophagy plays a positive role in maintaining proper endometrial function and is important for the decidualization process of human ESCs [108]. Knockdown of key autophagy-related proteins, ATG7 and ATG5, impairs the transition from ESCs to DSCs during decidualization [109]. Insufficient autophagy in DSCs can also disrupt the residence of dNK cells, leading to an increased risk of embryo loss. However, this can be reversed by low doses of rapamycin, which is known to induce autophagy [79].

Ferroptosis in ESCs can contribute to the production of angiogenic, inflammatory, and growth-promoting cytokines like vascular endothelial growth factor A and IL8, promoting angiogenesis in adjacent lesions and ultimately leading to the development of endometriosis [110] (Fig. 3B). Moderate apoptosis and autophagy are conducive to decidualization, however, the direct impact of necroptosis and ferroptosis on DSCs is still unclear and requires further investigation.

## Effect of RCD on immune microenvironment

The maternal-fetal interface acts as a physical barrier between the fetoplacental unit and maternal blood, protecting against microbial invasion. While some viral infections can be transmitted vertically from pregnant women to the fetus [111], others may not have the same capability [112]. Interestingly, primary human placental trophoblast cells have demonstrated high resistance to viral infections, such as VSV. They secrete placenta-specific miRNAs via exosomes, which confer viral resistance to recipient cells. Autophagy induced by miRNAs target cytoplasmic viruses, leading to their degradation in lysosomes [113]. In contrast, maternal infection with the Zika virus (ZIKV) can trigger apoptosis and result in neonatal microcephaly and neurological disorders [114, 115]. Treatment with chloroquine has shown significant improvements in adverse outcomes by inhibiting ZIKV RNA replication and blocking ZIKV-induced autophagy [116]. In the presence of a caspase inhibitor, porcine parvovirus induces non-apoptotic cell death linked to autophagy [117]. The activation of autophagy by porcine parvovirus promotes viral replication in porcine placental trophoblasts [118]. Hence, the role of autophagy in viral resistance varies depending on the virus type and may also involve other yet undiscovered forms of cell death.

Apoptosis plays a significant role in maintaining fetal immune tolerance at the maternal-fetal interface [119, 120]. In pregnancies with fetal growth restriction, the expression levels of FasL are reduced in decidual cells, indicating a relationship between apoptosis and diminished immune privilege [121]. Dysregulation of necroptosis and pyroptosis leads to cell membrane damage and release of cellular contents, triggering an inflammatory response and immune activation at the maternal-fetal interface [122, 123]. Although the deletion of gasdermin E (GSDME) does not impact the development or immune system of mice [124], it has been observed that pyroptosis by activating GSDME affects the innate immune system and leads to placental damage during ZIKV infection [125]. Thus, RCD modulates immune cells to influence the immune microenvironment at the maternal-fetal interface, playing a role in viral infections.

## RCD in different pregnancy complications

RCD is influenced by various factors, including viruses, bacteria, drugs, nutrient metabolites, and exposure to environmental pollutants [126–129]. RCD disorders in trophoblasts and decidual cells can affect their normal physiological function or impact surrounding cells through cytokine secretion, thereby resulting in abnormal placentation, reduced fetal growth, and pregnancy complications.

### Preeclampsia

The pathogenesis of preeclampsia (PE) remains controversial, but both clinical and pathological studies suggest that abnormal placental development, specifically inadequate trophoblast cell invasion and spiral artery remodeling, plays a central role [130, 131]. RCD has been implicated in the occurrence and progression of PE.

Excessive apoptosis is closely related to PE, and can be partly reversed by treatments like aspirin, cyclosporine A and resveratrol [132–134]. Pyroptosis in trophoblast cells promotes the occurrence and development of PE [135]. Some studies have confirmed that pyroptosis occurs in trophoblasts of women with early-onset PE, as evidenced by elevated levels of active caspase-1, GSDMD, IL-1β, and IL-18 [42, 136, 137]. Placental pyroptosis is a critical event leading to the release of inducible factors into the maternal circulation, which can result in severe sterile inflammation and the pathology associated with early-onset PE [42]. Additionally, elevated levels of miR-124-3p have been shown to promote pyroptosis by inhibiting the expression of placental growth factor (PLGF) and increasing intracellular ROS [138]. In the placentas of preeclampsia patients, trophoblast cells undergo necroptosis, as evidenced by increased expression levels of RIPK1 and RIPK3 and phosphorylation of MLKL [41, 139]. Thus, apoptosis, necroptosis, and pyroptosis are associated with the placental pathophysiology of PE.

Compared to healthy controls, placental iron content is increased in patients with PE, but levels of the iron exporter ferroportin 1 and GSH, GPX4 activity and serum selenium levels are significantly decreased [140]. Inhibition of GPX4 in HTR8 cells leads to ferroptosis, while knockout of GPX4 in mice results in embryonic lethality [141]. The upregulation of miR-30b-5p in the placenta can induce ferroptosis by reducing GSH levels and increasing the labile iron pool, thus contributing to the development of PE [60]. Analyzed gene expression from GEO database have found that ferroptosis is implicated in the pathogenesis of early-onset PE [142]. SRXN1 and NQO1 are two newly identified critical molecules that may be key ferroptosis-related proteins [143]. Elabela and calcium-independent phospholipase A2 protein (PLA2G6) could inhibit ferroptosis in trophoblast cells to alleviate preeclampsia [144]. *miR-2115-3p* decreases the expression level of glutamic-oxaloacetic transaminase, which further inhibits hypoxia-triggered ferroptosis [145]. These findings indicate ferroptosis involved in the development of PE and suggest potential therapeutic targets.

Under physiological hypoxic conditions, hypoxia-inducible-factor-1α-induced autophagy is an energy source for the invasion of EVTs [146, 147]. However, dysregulation of autophagy is involved in the pathophysiological processes of PE. Increased levels of BECN1 have been reported in the placentas of women with PE [148]. The increase in LC3-II and decrease in p62 levels in the placenta of women with hypertensive disorders also indicate autophagic activation [149]. Excessive autophagy activation induced by nanoparticles or oxidative stress in trophoblast cells impairs trophoblast invasion and the placental vasculature, potentially contributing to the development of preeclampsia [54, 150]. During pregnancy, the shedding of trophoblasts into the maternal circulation facilitates the generation of tolerogenic embryonic antigen-specific T cells by supplying antigens to the maternal immune system [151]. But in some cases, cell debris or placental factors from syncytiotrophoblast released into the maternal circulation may activate endothelial cells or induce an inflammatory environment, leading to preeclampsia [152, 153]. This suggests that fetual-derived cell death plays a role in maternal regulation, but the specific RCD causing the death of syncytial trophoblast cells has not been further studied. Thus, RCD is involved in the pathogenesis of preeclampsia.

### Gestational diabetes mellitus

Gestational diabetes mellitus (GDM) is a serious pregnancy complication that arises when women develop chronic hyperglycemia during pregnancy without a prior diabetes diagnosis. The pathogenesis of GDM is closely linked to ferroptosis. The decreased glutathione level, impaired iron transport and increased lipid peroxidation of BeWo cells cultured in hyperglycemia environment suggested that ferroptosis occurred under these conditions [154]. Furthermore, high glucose levels activate autophagy-dependent ferroptosis by affecting the AMPK-mTOR pathway and reducing GPX4 levels in trophoblastic cells [155].

Autophagy is significantly increased in patients with GDM, as evidenced by higher levels of autophagy-related proteins like LC3-II and Atg5, and lower levels of p62 in placenta [156]. In cultured HTR8 cells, elevated glucose levels enhance autophagy while reducing cell proliferation and invasion, these effects can be reversed by knocking down ATG5 [157]. A recent study found that the orphan nuclear receptor NUR77 is abnormally upregulated in the placenta of mice with GDM, and it inhibits insulin sensitivity by promoting the expression of Beclin 1 and the LC3II/LC3I ratio in HTR8 cells [158]. Additionally, the hepatocytes of GDM offspring show a significant increase in intrahepatic autophagosomes compared to normal offspring [159].

Direct roles of necroptosis and pyroptosis at the maternal-fetal interface in GDM have not been reported; however, there are indications that they may be involved in the regulation of GDM. Activation of aldehyde dehydrogenase 2 (ALDH2) prevents necroptosis in a model of primary cardiomyocyte injury induced by high glucose levels [160]. Notably, placentas from GDM pregnancies exhibit reduced expression levels of ALDH2 [161]. Furthermore, elevated secretion of active caspase-1 and mature IL-1β has been observed in the adipose tissue of GDM patients, suggesting the possibility of pyroptosis [162]. Further investigation is needed to understand the impact of necroptosis and pyroptosis at the maternal-fetal interface in GDM.

### Recurrent spontaneous abortion

Spontaneous abortion is one of the most common pregnancy complications. Recurrent spontaneous abortion (RSA) is defined as the occurrence of two or more pregnancy losses before 20–24 weeks of gestation. Though the etiology of RSA remains unclear, dysregulation of RCD is one reason.

Abnormal apoptosis of the DSCs, decidual immune cells and trophoblasts may cause spontaneous abortions. Studies have shown increased apoptosis in the placenta and decidua of women who have experienced spontaneous abortions [163, 164]. Elevated levels of pro-inflammatory cytokines can affect the expression of CCL28 and its receptors, leading to DSC apoptosis and ultimately resulting in spontaneous abortion [165]. Huang et al. found that reduced IDO^+^ decidual macrophages promotes trophoblast cell apoptosis and increasing the risk of abortion [84]. dNK cell-derived granulysin regulate EVT cells apoptosis by granulysin, suggesting that dNK cells are involved in miscarriage [166]. Abnormal trophoblast apoptosis, caused by factors such as NO content, or *miR-184*, may also contribute to the development of recurrent spontaneous abortion [86, 167, 168].

The relationship between altered autophagy and RSA is still ambiguous [169]. Abnormal distribution of autophagosomes has been found in the villi of RSA patients [170, 171]. Inhibited autophagy in trophoblasts increases the cytotoxicity of NK cells, impairs trophoblast invasion, and eventually leads to abortion [50]. In therapeutic studies, Rapamycin has been shown to significantly promote DSC autophagy and NK cell residence to improve embryo resorption [79, 87]. However, a study showed that HMGB1 and autophagy are significantly higher in abortive villus tissues. HMGB1 is involved in LPS-induced inflammation through autophagy and may be involved in the pathogenesis of miscarriage [172]. Paradoxical changes in autophagy may occur due to its compensatory function under certain conditions or its regulation by multiple upstream factors.

Overexpression of HMGB1 at the maternal-fetal interface of RSA patients is not only linked to autophagy but also to pyroptosis. Zou et al. first found that HMGB1 promotes RSA by activating the RAGE/TLR2/TLR4-NF-κB pathway at the maternal-fetal interface [173]. Subsequently, they reported that decidual macrophages secrete a large amount of HMGB1, which activates pyroptosis, leading to sterile inflammation and promoting the occurrence and progression of RSA [83]. In experiments using poly(I:C), a viral infection mimic, it was found that necroptosis is induced in DSCs, resulting in mouse abortion. Mice deficient in MLKL exhibited milder pathological changes in the uterus, indicating the involvement of necroptotic signals in the regulation of dsRNA-triggered abortion [107]. Therefore, various forms of regulated cell death play a role in the development and occurrence of RSA.

## Discussion

The purpose of this review was to provide an updated perspective on RCD in trophoblast cells, DSCs and decidual immune cells at the maternal-fetal interface, and its effects on cell function, fetal development, and pregnancy-related disorders. We found that many factors can trigger RCD, including bacterial and viral infections, exposure to environmental pollutants, drug side effects, disruptions in nutrient metabolism, cytokine secretion, and hormonal changes. Interestingly, different stimuli can induce the same type of cell death, while the same stimulus can affect different types of RCD. Notably, SIRT3 deficiency leads to resistance to autophagy-dependent ferroptosis and is implicated in the development of preeclampsia by promoting necroptosis, highlighting the importance of SIRT3 as a potential target for RCD regulation.

Trophoblasts undergo multiple forms of RCD, including apoptosis, autophagy and necroptosis, which play crucial roles in trophoblast differentiation, invasion and remodeling of uterine spiral arteries. Additionally, RCD of DSCs is highly involved in decidualization, facilitating the infiltration and residence of dNK, macrophages and T cells, thereby promoting endometrial receptivity and preventing viral invasion.

In addition, RCD serves as a mode of communication between different cells, maintaining the immune microenvironment at the maternal-fetal interface. Inadequate autophagy of DSCs leads to inadequate residence of NK cell and macrophage, while impaired autophagy in trophoblast cells increases NK cell toxicity, disrupting immune homeostasis. By regulating the differentiation of decidual macrophages, ferroptosis, pyroptosis and autophagy can affect the immunological milieu. Trophoblasts exploit exosomes to transfer placenta-specific miRNAs then induce autophagy of other cells and provide them with antiviral properties. Additionally, during syncytial fusion, apoptotic component released into the maternal circulation, facilitate interaction with immune cells and reduce the risk of fetal rejection.

The impact of apoptosis and autophagy on trophoblasts, placental development and decidualization is complex and has dual effects. The differentiation and fusion of mononucleated cytotrophoblasts into syncytiotrophoblasts relies on moderate apoptosis. The remodeling of the uterine spiral artery requires trophoblasts to secrete cytokines that induce moderate apoptosis in smooth muscle and endometrial cells. Appropriate autophagy also plays an important role in trophoblast invasion of the decidua. However, superfluous autophagy leads to adverse pregnancy outcomes. Moreover, TNFα-primed epithelial necroptosis in the uterine epithelium positively affects successful embryo invasion. The release of cellular contents during necroptosis can trigger changes in the surrounding cells at the implantation site, promoting maternal remodeling of the uterus and ensuring healthy fetal development. On the other hand, poly(I:C)-triggered necroptosis in decidual stromal cells contributes to abnormal pregnancy. The potential roles of pyroptosis and ferroptosis during pregnancy require further investigation. Therefore, maintaining a balance between inhibiting and inducing RCD is crucial for successful pregnancy and normal fetal development.

Other types of RCD like cuproptosis discovered by Peter Tsvetkov and colleagues in 2022 involves the excess intracellular Cu(II) being transported to the mitochondria via ion carriers, resulting in protein toxicity stress and ultimately leading to cell death [174]. It has been found that CuO Nanoparticles can induce cuproptosis and ferroptosis in trophoblast cells and can be used for female contraception [175].

During embryo implantation, decidual macrophages can clear apoptotic cells through efferocytosis. Trophoblasts can influence the metabolic reprogramming and efferocytosis of macrophages [176]. In addition, there may be different efferocytosis in macrophages, and the mode of death of the phagocytosed cells may also determine the function of efferocytosis, imbalance in efferocytosis induced by metabolic reprogramming of macrophages is associated with pregnancy loss [177].

Although PANoptosis has not been reported in the development of the maternal-fetal interface, we believe it plays a role in this process. Because PANoptosiss is characterized by selecting the best pathway among multiple different types of cell death pathways to eliminate eliminated cells, maintaining tissue and organ homeostasis [17]. In the biological evolution, the body is bound to adopt the optimal mode of cell death regulation in reproduction.

## Conclusions and future perspectives

This study describes several common RCD patterns and their impact on the function of trophoblast cells, DSCs and decidual immune cells at the maternal-fetal interface, as well as in pregnancy-related disorders. Autophagy and apoptosis are involved in trophoblast differentiation and invasion and spiral artery remodeling under physiological conditions, they also play an important role in the regulation of immune cells and decidual cells. Although necroptosis is beneficial to trophoblast invasion, current studies have shown that necroptosis, like ferroptosis and pyroptosis, plays a more negative role at the maternal-fetal interface. It remains unclear whether ferroptosis and pyroptosis positively regulate normal pregnancy, probably due to the infrequent occurrence of these modes of cell death under physiological conditions. Additionally, our understanding of the mechanisms underlying these RCD is still limited, and there is a lack of specific detection indicators.

The inhibition of RCD has emerged as a potential strategy for treating a variety of human diseases. The anti-cancer drug vemurafenib can serve as an effective RIPK1 antagonist, effectively inhibiting necroptosis and the occurrence of related diseases [178]. The protective effect of mannose in inhibiting pyroptosis of normal gastrointestinal cells has been preliminarily confirmed in clinical trials involving gastrointestinal cancer patients undergoing chemotherapy [179]. However, to date, no RCD inhibitors have been approved for clinical use. Encouragingly, the use of RCD inhibitors or agonizts, such as necrostatin-1 and rapamycin, has shown promise in ameliorating adverse pregnancy [71, 79].

Therefore, a series of basic and translational research on potential new therapeutic targets and signaling pathways in various RCD pathways is of significant importance for the discovery of new drug targets, drug design and structure optimization, and mechanism of action studies. Considering that RCD can affect the majority of cells in the maternal-fetal interface, it is also worth thinking about how to target a certain type of cells to play a better role when drug therapy is based on RCD. Well-designed studies are necessary to validate the role of RCD at the maternal-fetal interface and delineate the network of associated regulatory mechanisms. This will facilitate the development of potential treatment strategies in clinical practice.
